# Mast Cells: Key Players in the Shadow in Oral Inflammation and in Squamous Cell Carcinoma of the Oral Cavity

**DOI:** 10.1155/2016/9235080

**Published:** 2016-10-26

**Authors:** Pusa Nela Gaje, Raluca Amalia Ceausu, Adriana Jitariu, Stefan Ioan Stratul, Laura-Cristina Rusu, Ramona Amina Popovici, Marius Raica

**Affiliations:** ^1^Department of Microscopic Morphology/Histology, Angiogenesis Research Center Timisoara, “Victor Babes” University of Medicine and Pharmacy, Timisoara, Romania; ^2^Department of Periodontology, “Victor Babes” University of Medicine and Pharmacy, Timisoara, Romania; ^3^Department of Technology of Materials and Devices in Dental Medicine, “Victor Babes” University of Medicine and Pharmacy, Timisoara, Romania; ^4^Department of Preventive Dental Medicine, “Victor Babes” University of Medicine and Pharmacy, Timisoara, Romania

## Abstract

Although mast cells (MCs) have been discovered over 130 years ago, their function was almost exclusively linked to allergic affections. At the time being, it is well known that MCs possess a great variety of roles, in both physiologic and pathologic conditions. In the oral tissues, MCs release different proinflammatory cytokines, tumor necrosis factor alpha (TNF-*α*), that promote leukocyte infiltration in various inflammatory states of the oral cavity. These cells play a key role in the inflammatory process and, as a consequence, their number changes in different pathologic conditions of the oral cavity, like gingivitis, periodontitis, and so on. MCs also represent a rich source of proteases, especially of mast cell tryptase and chymase, which directly degrade the extracellular matrix through their proteolytic activity and thus indirectly stimulate angiogenesis and facilitate invasion and metastasis. It may be stated that mast cells could have an impact on primary tumor development, progression, and metastases in oral squamous cell carcinoma. By understanding the role of mast cells in the pathogenesis of different inflammatory and tumor diseases of the oral cavity, these cells may become therapeutic targets that could possibly improve the prognosis and survival of these patients.

## 1. Introduction

Mast cells have been described for the first time by Ehrlich in 1878 [1, 2]. He used the term “mastzellen,” a German term that refers to feeding, in order to describe these cells. Ehrlich also noted the association between mast cells and inflammation, blood vessels, and nerves. He also observed an increase in the number of mast cells in the tumor stroma, particularly in carcinoma. Mast cells are currently regarded as potent effector cells of the immune system. The role of mast cells in allergic diseases, anaphylaxis, and autoimmunity has been well documented. However, their role in the pathogenesis of oral pathologies is still debatable. Hence, the present review article aims to explore the role of mast cells in the initiation and progression of oral pathological conditions, such as oral inflammatory lesions and oral squamous cell carcinomas.

### 1.1. Phenotype and Location

Despite the fact that all mast cells derive from a common precursor and have granular cell morphology, they appear to be extremely heterogeneous in terms of phenotype and function [3]. Two mast cell phenotypes have been described in rodents: CTMCs (connective tissue MCs) and the MMCs (mucosal MCs) which differ through their localization, mediator content, and response to different stimuli. The two phenotypes have been characterized based on their heterogeneous expression of proteases, including mast cell tryptase and chymase. CTMCs contain tryptase and chymase, while MMCs contain only chymase [4]. Human mast cells are also split into two types depending on their content in proteases. Thus, some mast cells contain only tryptase in their granules (MC_T_), while others contain both tryptase and chymase (MC_TC_) [5]. Regarding their localization, MC_T_ type is mainly found in the lung interalveolar septae and in the small intestine mucosa, while MC_TC_ are predominant in the skin and in the intestinal submucosa.

Mast cells are located at the host-environment interface, in the immediate vicinity of blood vessels, lymphatic vessels, nervous fibers, but also in the immediate vicinity of the cells belonging to the immune system [6]. Due to this strategic localization, mast cells act as germ invasion sentinels, but are also able to hastily respond to any change that occurs in the surrounding microenvironment due to their interactions with other cells implicated in the physiologic and immunologic response.

Mast cells are found in all connective tissue types of the oral cavity, including the periodontal ligament, the dental pulp, and the gingiva [7, 8]. Their presence in the dental pulp is difficult to detect due to the fact that the tissue lesions that occur during dental pulp prelevation request mast cell degranulation, which makes it difficult to evidence degranulated mast cells by means of classic histochemical staining. In contrast, immunohistochemical staining methods and electron microscopy are able to demonstrate mast cell degranulation [9]. In the gingiva, mast cells have been observed in both normal and inflammatory conditions, even in experimental gingivitis [10]. At the level of the oral mucosa, mast cells are preferentially distributed adjacent to the microvessels, in the immediate vicinity of the basement membrane. Except for their presence in the normal oral mucosa, mast cells have been associated with a wide range of oral affections, such as periapical lesions [11], gingivitis [12], odontogenic cysts [13], pyogenic granulomas, and lichen planus [14].

### 1.2. MCs: What Do They Look Like?

When viewed by light microscopy, human MCs usually appear as round or oval in shape with a diameter ranging between 8 and 20 *μ*m depending on the organ in which they are studied. Their nucleus is round or ovoid and their cytoplasm contains numerous secretory granules that are metachromatic with toluidine blue. Besides metachromatic granules, the cytoplasm also contains a few mitochondria, short profiles of the rough endoplasmic reticulum, and numerous free ribosomes [15].

When examined in electronic microscopy, mast cell granules, from the oral mucosa and from the skin, show a complex ultrastructure, possessing an amorphous region which is situated next to the crystalline region. The crystalline region presents a varied configuration and, thus, three distinct mast cell populations have been identified. Accordingly, mast cells located in the deep connective tissue are round or oval in shape and have well defined cell borders. The nucleus cannot be observed because it is often obscured by cytoplasmic granules. These mast cells were named “intact cells” [16]. MCs located in the superficial zone of the connective tissue and those situated in the vicinity of blood vessels are flattened or irregular in shape, and their cytoplasm presents a granular aspect. The cell borders are not well defined, and the nucleus is partially noticeable. These cells have been named “spreading cells” [17]. The third cell type is represented by the degranulated mast cells that have been observed as cell infiltrates [18].

### 1.3. MCs Mediators

Mast cell granules contain a great variety of mediators, which may be grouped into two categories: preformed and* de novo.* Preformed mediators are represented by tryptase, chymase, cathepsin G, histamine, heparin, serotonin, IL-16, and TNF-*α*. Mediators synthesized following mast cell activation are represented by interleukins IL-1, IL-3, IL-4, IL-5, IL-6, IL-8, IL-10, IL-13, and IL-16, platelet activating factor (PAF), RANTES, MIF-1 alpha (macrophage inhibitory factor) and arachidonic acid metabolites, prostaglandin, and leukotriene C4 (LTC4) [18, 19]. Besides conventional mediators and cytokines, MCs are able to secrete some growth factors, like vascular endothelial growth factor, fibroblast growth factor, and nerve growth factor, that contribute to homeostasis and development and are also involved in some pathological conditions. Not all MCs synthesize and secrete all mediators; the profile of each subpopulation strongly depends on the localization, pathological condition, and functional status.

### 1.4. Mast Cell Degranulation

Degranulation of mast cells releases proinflammatory mediators such as tryptase, chymase, heparin, histamine, TNF-*α*, MMPs, basic fibroblast growth factor (bFGF), various interleukins (IL-3, IL-4, IL-5, IL-6, IL-8, IL-10, IL-13, and IL-16), and cytokine RANTES [20, 21]. Degranulation is induced by various stimuli like IgE receptors, chemokines, neuropeptides, and other physical stimuli.

Cytokines and chemokines produced by mast cells include classical proinflammatory mediators, as well as cytokines that are associated with anti-inflammatory or immunomodulatory effects, such as IL-10 and TGF-*β* (transforming growth factor-*β*). Although frequently described as a source of T helper 2- (T_H_2-) type cytokines, including IL-4, IL-5, and IL-13, mast cells can also produce T_H_1-type cytokines, such as IFN-*γ* (interferon *γ*), IL-12, and IL-18 [22].

RANTES (regulated upon activation, normal T cell expressed and secreted) are a member of chemokine family produced by various cells, such as activated T-lymphocyte, bronchial epithelial cell, oral keratinocytes, and mast cell. RANTES secreted by activated T cells attract mast cell and stimulates degranulation [23].

Tryptase is the most abundant serine proteinase stored in mast cell granules. It promotes inflammation and tissue remodeling and is considered an important angiogenic factor [24].

Therefore, mast cells release low molecular weight substances, such as histamine, as well as cytokines and chemokines that may exert profound effects in oral inflammation and cancer.

## 2. Mast Cell Gameplay in Oral Inflammation

Well known for their role in allergic reactions, mast cells are also involved in immunity and inflammation. Increasing evidence indicates that mast cells are critical for the pathogenesis of inflammatory diseases [25], such as atopic dermatitis, psoriasis [26, 27], arthritis [28], and multiple sclerosis [29]. Gene analysis made on human mast cells activated by IgE shows an overexpression of several genes, mainly those that are linked to inflammation [30]. Proteases liberated by mast cells may act on plasma albumins in order to generate histamine release [31, 32] that may favor mast cell activation and inflammation. Proteases may also stimulate protease activated receptors (PAR) inducing the spread of the inflammatory process [33, 34]. Unlike allergic reactions, inflammatory processes are characterized by rarely degranulated mast cells. The only way to explain the implication of mast cells in the basic nonallergic processes would be the “selective” release mediators without degranulation [35].

Inflammation is a critical process in the oral cavity, gingival, and dental pulp inflammations, especially, as it may be observed in periodontitis and in the structure of the carious tooth. However, the cellular process implicated in oral inflammation is not yet well defined. Chronic inflammation is a useful tool that permits the investigation of mast cell reaction as a response to minimal stimuli, and their local response is usually associated with an accumulation of lymphocytes, macrophages, and plasma cells.

Mast cell degranulation is followed by the release of mediators such as proinflammatory metabolites of arachidonic acid, proteases, histamine, proteoglycans, chemokines, and growth factors. Histamine promotes platelet cell adhesion through mobilizing the adhesion molecule P-selectin (CD62P, GMP-140) stored in the Weibel-Palade corpuscles located at the luminal surface of endothelial cells [36]. Hence, literature includes studies that directly proved the release of histamine from mast cells in the oral mucosa [37]. Of all the cytokines secreted by MCs, tumor necrosis factor alpha is of special interest due to the fact that it is associated with the inflammation of the oral cavity [38].

Conflicting results are available in literature concerning mast cell density in oral inflammatory lesions [39]. Some of them show an increase in the density of mast cells, while others show a decrease or even a lack of these cells in inflammatory conditions. Thus, the lack and the decrease in mast cell density have been reported in acute necrotizing gingivitis and marginal chronic gingivitis compared to the normal gingiva [40]. However, an increased mast cell density has been reported in the case of gingival hyperplasia [41]. An increase in the average number of mast cells was also observed in reactive inflammatory conditions, such as inflammatory hyperplasia, in the granulation tissue, and in gingivitis [42], as well as in vascular states, such as hemangiomas [43], thus suggesting that MCs may be key players in the recruitment of inflammatory cells and in angiogenesis. In the case of periodontal disease, the increase in mast cell density was reported by several authors, but results are contradictory, maybe partially due to the different methods used to quantify MCs and, on the other hand, due to a relatively low number of cases [44, 45]. Neither of these studies correlated the number of mast cells with the density and distribution of the inflammatory infiltrate.

Back in 2010, Farahani et al. compared the density of mast cells in different reactive oral lesions such as irritative fibroma, fibrous inflammatory hyperplasia, peripheral granuloma with giant cells, and peripheral bone forming fibroma. They observed an increase in the number of mast cells in reactive lesions, compared to the normal gingival tissues. Mast cells have significantly decreased in the peripheral granuloma with giant cells group compared to the fibrous inflammatory hyperplasia and the peripheral bone forming fibroma. The result of this study suggests that MCs may play an important role in collagen synthesis and in the variations of the microscopic characteristics of the soft tissue reactive lesions in the oral cavity [46].

Some evidence concerning the oral cavity suggested that neurogenic inflammation involved the participation of MCs. These may represent a critical factor, probably due to their disposition in the strategic points, like the vicinity to the small blood vessels and nervous fibers which often contain the P substance (SP). Algia models, of reversible or irreversible pulpitis, simply suggest the complexity of the interaction between the neuroinflammatory mediators from the dental pulp. In the dental pulp, as well as in the periapical area, neuropeptides and cytokines modulate the vascular response, the increase in the vascular permeability, and leukocyte migration. In periapical granulomas TNF-positive MCs were observed surrounding the blood vessels and the SP immunoreactive nervous fibers. Thus, through generating an increased number of proinflammatory mediators, MCs may serve as bridges between the nervous, immune, and endocrine systems in inflammatory lesions of the dental pulp. This role was consolidated by other proofs regarding MCs implications in inflammatory reactions [47].

The demonstration of mast cell presence in the dental pulp was quite the challenge due to the fact that the tissue lesions that occur during dental pulp removal require degranulation, and, on the other hand, it must be stated that the dental pulp also contains progenitor MCs, which can exclusively be identified through their granules.

In the human dental pulp tissue that has been subject to inflammation, high concentrations of TNF-*α* have been detected. These concentrations decrease when the inflammation of the pulp progresses towards necrosis, while the lowest concentration is found in the healthy pulp tissue [48]. Similar discoveries were found in rat teeth following pulp inflammation [49]. Thus, the literature data suggested that the source of TNF-*α* may be oral MCs granules, which contain TNF-*α*. This factor is released upon degranulation process. Some data suggest that mast cell histamine, which is a strong vessels dilator and mediator of vascular permeability, may play a role in the initiation of dental pulp inflammation [50]. P substance, on the other hand, may mediate mast cell degranulation following MCs activation at the level of the dental pulp [51]. Bacterial invasion of the pulp during caries formation may also provoke MCs activation. In 2009, Karapanou et al. came up with a hypothesis that MCs activation may take place via the neuropeptides that are locally released in the pulp, and, afterwards, proinflammatory mediators that are liberated from the mast cell granules may participate in the inflammatory process of the dental pulp and may serve as diagnostic markers for inflammatory teeth [47]. Hence, it may be stated that local activation of MCs is a frequent event and even a condition for inflammation occurrence.

In the oral cavity, the gingiva contains the highest number of MCs and, like in many other locations of the human body, the intrinsic role of mast cells still remains elusive in both normal and pathologic conditions. A significant increase in mast cell density has been found in gingival inflammation, but no explanation regarding their implication in the maintenance and progression of the inflammatory process has been revealed [52]. Moreover, there are no data on the relationship between MCs and the density of the inflammatory infiltrate.

A recent study carried out by Huang et al. [53] demonstrated a correlation between MCs degranulation and periodontitis severity. The authors observed that the density of the positive-tryptase degranulated MCs is significantly higher in severe periodontitis, compared to mild periodontitis and compared to the normal tissue. However, it was not possible to conclude whether degranulation had been induced by the inflammatory infiltrate or if it induces the accumulation of inflammatory cells. In contrast, another study reports a decrease in MCs density (MCD) in cases of severe periodontitis. The multiple functions of mast cells represent a possible explanation for the decreasing of the mast cell density in the severe periodontitis [54].

In periodontal disease, MCs are implicated in the recruitment of inflammatory cells and in the formation of new blood vessels. MCs implication in inducing and maintaining angiogenesis and lymphangiogenesis has already been demonstrated in clinical and experimental conditions. This aspect is supported through the expression of the vascular endothelial growth factor (VEGF) by the epithelial cells of the oral mucosa, but also by the increased microvessel density in pathologic conditions [55].

At the time being, it is accepted that mast cells play a specific role in the pathophysiology of numerous affections, and, through the release of granule mediators, they contribute to the tissue lesions and inflammation. Recent data showed that inflammation is a critical component for tumor progression [56].

## 3. Mast Cells in Oral Squamous Cell Carcinoma

Squamous cell carcinoma is a malignant tumor that derives from covering epithelium, including those of the oral mucosa. This tumor type represents approximately 90% of all malignant tumors from the oral region and remains a serious oral health problem at global level [57]. Thus, Huang et al. showed that squamous cell carcinoma in men is more often located in the oral mucosa, probably due to frequent exposure to carcinogenic agents [58].

Angiogenesis or neovascularization is an important component of several biological processes, in both physiologic and pathologic conditions, such as neoplasms [59]. Angiogenesis is known to aid progression and metastasis in different malignant tumors including the tumors of the oral cavity [60, 61]. MCs stimulate neovascularization through the release of angiogenic factors, such as VEGF, or different substances with angiogenic properties, such as tryptase, IL-8, tumor necrosis factor (TNF), basic fibroblast growth factor (bFGF), heparin, and histamine. Mast cell heparin induces vessel proliferation and increases basic FGF half-life, which is a strong angiogenic substance, thus promoting tumor angiogenesis and facilitating the local invasion. Interleukin-1 (IL-1) leads to epithelial proliferation [61, 62].

Mast cells are also a rich source of proteases, especially tryptase and chymase, which directly degrade the extracellular matrix through their proteolytic activity and indirectly stimulate angiogenesis and facilitate invasion and metastasis through extracellular matrix remodeling. Also, the matrix metalloproteinases produced by MCs may contribute to extracellular matrix degradation. Thus, MCs may have an impact both on the development of the primary tumor and on the following tumor progression and metastases in squamous cell carcinomas of the oral cavity.

Besides the role of MCs in maintaining homeostasis and inflammation, several studies described an association between mast cell density and different malignant tumors, amongst which the oral squamous cells carcinoma is considered. The presence of MCs at the periphery of the tumor areas was reported for the first time by Westphal in 1891, and since then this observation was confirmed by numerous researchers [63].

The increase in mast cell density is associated with poor prognosis in different malignant tumors, indicating their role in tumor progression [64, 65]. Recent data suggest that mast cell accumulation at the periphery of tumor areas and the release of proangiogenic factors may represent a tumor-host interaction that probably favors tumor progression [66, 67].

Currently, the exact functional relevance of mast cells accumulated around malignant tumors is being studied [68]. Although it has been suggested that mast cells are important players in connective tissue diseases and in inflammatory allergic affections, the functional significance of mast cells in different tumors is not yet fully understood [69]. Oral squamous cell carcinoma is usually associated with chronic inflammation, angiogenesis, and immune reactions. In experimental model of squamous carcinogenesis, an increased number of MCs during the transition from dysplasia to carcinoma was found. This is why the evaluation of MCs role in these tumors became a necessity. The immunological and inflammatory responses that occur in the tumor microenvironment have been debated by several studies. While immune-inflammatory cells may participate in the local tumor immunity, reducing tumor invasion, these cells may also contribute to tumor progression and metastasis. Under these circumstances, MCs play an important role in host defense, due to their location at the host-environment interface and due to their capacity to store preformed mediators that may contribute to immune regulation, matrix degradation, and angiogenesis [70].

In oral squamous cell carcinomas, MCs have been identified through several histochemical and immunohistochemical methods. The morphologic evaluation of the relationship between the mast cell and the blood vessels may be done through the identification of the highly specific markers for the mast cell and for the endothelial cell by means of double immunostaining. The double immunoreaction for mast cell tryptase and CD31 or CD34 is relatively simple and accurate, but similar results, maybe even more complete from a functional point of view, are obtained by using safranin alcian blue/CD34 on the same section. The histochemical method is done after immunohistochemistry, and stained sections are useful to evaluate both microvessel density and mast cell density on the same microscopic fields. The advantage of this method consists in the fact that it identifies three different mast cell populations, an aspect that is not relevant using only anti-tryptase antibodies, which is a constitutional enzyme that is found in all mast cell types. The density of the mast cell population is calculated through the application of the same principles, similar to the procedure used to estimate microvessel density. The results obtained by using this method correlate with those found in literature regarding the increased number of mast cells observed in malignant tumors. In the squamous cell carcinoma of the lip, the increased microvessel density correlated with mast cell density, and this suggests their cooperation in tumor angiogenesis, possibly through VEGF secretion [71].

Costa et al. [72] observed that the mast cell density in squamous cell carcinomas of the lip was significantly higher as compared to the normal mucosa of the lips. The impact of the increase in MCs density and angiogenesis in SCC of the lip may reflect an important change in the tumor microenvironment during squamous photocarcinogenesis.

Several other studies showed that MCs density is significantly higher in actinic cheilitis [73] and in the squamous cell carcinoma of the lip [74] and is associated with favorable effects for the tumor [61, 74]. Moreover, in an experimental model of carcinogenesis, mast cell density has been associated with carcinoma development through the regulation of angiogenesis during the premalignant and malignant stages of squamous epithelial carcinogenesis [75, 76]. On the other hand, some studies suggest an antagonist effect of mast cells on the tumor, due to the fact that mast cell deficient mice present an increased tumor incidence following treatment with a carcinogenic agent [77].* In vivo* studies showed a sequential infiltration of mast cells and their degranulation during carcinogenesis in the oral squamous cell carcinoma and demonstrated the strict correlation between mast cell activation and different phases of hyperkeratosis, dysplasia, in situ carcinoma, and oral invasive carcinoma [78].

Michailidou et al. [67] evaluated the relationship between mast cells, angiogenesis, and histological progression from normal oral tissues to leukoplakia with different grades of dysplasia up to the oral squamous cell carcinoma. The authors observed an increase in the number of mast cells in leukoplakia with or without dysplasia compared to the normal tissue. A statistically significant correlation was found between mast cell density and microvessel density in leukoplakia with severe dysplasia and in the squamous cell carcinoma, mast cells being located in the areas that were provided with a rich vascular network. According to these results, a possible role of MCs in the progression of premalignant oral lesions into a squamous cell carcinoma is suggested.

On the other hand, Gomes et al. [79] studied the number of mast cells in 4 groups: normal oral mucosa (*n* = 6), actinic cheilitis with low grade dysplasia (*n* = 13), actinic cheilitis with severe grade dysplasia (*n* = 13), and squamous cell carcinoma of the lip (*n* = 15). The highest number of MCs per group was observed in the squamous cell carcinoma (40.1), followed by actinic cheilitis with low grade dysplasia (30.5), actinic cheilitis with severe grade dysplasia (28.6), and the normal oral mucosa (12.2). Significant differences have been noticed between the normal oral mucosa and actinic cheilitis with low grade dysplasia, but also between the normal oral mucosa and the squamous cell carcinoma of the lip. The increased MCs density observed in actinic cheilitis and in squamous cell carcinoma of the lip compared to the normal oral mucosa suggests their implication in the development of these lesions.

The progression of oral lesions from dysplasia to oral squamous cell carcinoma is characterized by an “angiogenic switch” that is associated with an increase in the neovascularization of the subepithelial lamina propria, which may be considered an indicator of malignant transformation. MCs represent a rich source for various angiogenic factors and, moreover, they secrete different proteolytic enzymes that might damage the extracellular matrix and create the space needed for blood vessel development [80].

Numerous studies evaluated the density of MCs in oral squamous cell carcinomas with different grades of differentiation. Thus, a study carried out by Kalra et al. [81] shows a decrease in mast cell density starting from well differentiated carcinomas to low differentiated ones. In contrast, the number of vessels increases starting from well differentiated carcinomas to low differentiated ones, showing an inverse relationship with the tumor grade. Through the evaluation of microvessel density they noticed a significant inverse correlation, however, between mast cell density and microvessel density. Thus, the low and moderate differentiated squamous cell carcinoma gained a strong angiogenic phenotype compared to the well differentiated carcinoma. In a similar manner, Sharma et al. [82] observed that microvessel and mast cell density are higher in moderate differentiated squamous cell carcinomas, compared to well differentiated carcinomas, supporting the hypothesis according to which MCs are probably implicated in the angiogenic switch. Hence, when compared to oral squamous cell carcinomas with different grades of differentiation, the low and the moderate differentiated carcinoma are known to be more aggressive and invasive and, in these cases, MCs may play a double role in promoting angiogenesis and invasion, while their cytotoxic function may be inefficient in such situations.

These results are in contrast with the study carried out by Iamaroon et al. [61] who noted that the microvessel and the mast cell density seem to increase along with disease progression from the normal oral mucosa, hyperkeratosis, and premalignant dysplasia to invasive oral SCC. Consequently, the authors suggest that the number of MCs may be used as indicators of disease progression. In another study regarding squamous cell carcinoma of the esophagus, Elpek et al. [68] noted a significant correlation between microvessel density and mast cell density. Increased values of these parameters were also associated with tumor progression. It is difficult to explain these discordant results regarding mast cell density and, thus, the hypothesis according to which the lower density observed in low differentiated oral SCC is possible due to massive mast cell degranulation may be stated, an aspect that makes their identification difficult.

Also, the study carried out by Oliveira-Neto et al. [83] shows mast cells density to be lower in oral squamous cell carcinoma and premalignant lesions compared to normal controls. They attributed it to migration failure of mast cells, which possibly reflect a modification in the microenvironment during tumor initiation and progression. Thus, Tomita et al. [84] suggest that there are two reasons that lead to such contradictory reports regarding the role of MCs. The cytotoxic functions of MCs that suppress tumor activities may be initially present when MCs infiltrate the tumor tissue. Despite these aspects, following infiltration, tumor cells may promote the angiogenic properties of MCs, suppressing their cytotoxic functions, thus leading to tumor angiogenesis. Consequently, the effect of MCs against tumor cells may depend on the concentration of mediators released by the MCs in the tumor microenvironment. Based on these findings, the authors consider that improvement of cytotoxic functions of MCs and the suppression of their angiogenic functions may lead to a new anticancer treatment strategy.

In 2010, Mohtasham et al. [85] compared mast cell density (MCD) and that of the microvessels (MVD) in the normal oral mucosa, dysplasia, and low and high grade oral squamous cell carcinoma. The authors observed a statistically significant increase of MCD and MVD between the normal oral mucosa and dysplasia, the normal oral mucosa and the oral squamous cell carcinoma, and dysplasia and the oral squamous cell carcinoma, but they did not observe any statistically significant difference between MCD and MVD in the low and high grade oral squamous cell carcinoma. The results of this study are consistent with the idea that MCs may promote tumor progression through the regulation of angiogenesis.

In their retrospective analytical study, Jahanshahi and Sabaghian [86] found a significant correlation between MVD and MCD in the normal oral mucosa and despite the high MCs density and the microvessels observed in oral SCC compared to the normal oral mucosa no significant correlation was noted between them.

In the head and neck region, Elpek et al. [68] showed that microvessel density is an accurate prognostic marker in squamous cell carcinoma of the esophagus. Additionally, mast cell density may play a role in angiogenesis of these tumors and may be responsible for their aggressive behavior [87]. The presence of MCs in the peritumoral areas of the oral squamous cell carcinoma was observed many years ago, but their role in tumor progression and metastasis still remains controversial.

## 4. Conclusion and Perspectives

Mast cells play a critical role in the development of inflammation at the levels of the oral mucosa and dental pulp, both during the early stages and during the transition from acute to chronic inflammation. Based on this concept, these cells may become a target for therapeutic agents that are capable of modifying their function and secretion and, thus, countering inflammation starting from its incipient phases.

Due to the fact that the role of MCs in the progression and metastasis of oral squamous cell carcinoma still remains a controversial issue, these cells must be further evaluated, using large samples that also include recurrent cases. Moreover, diverse factors that are linked to tobacco or associated tumors that may lead to an increase in mast cell density in oral squamous cell carcinomas must not be neglected. Moreover, the various factors (tobacco-related and tumor-related) that lead to an increase in MCs in oral squamous cell carcinomas need to be validated.

Hence, the profound understanding of the activation mechanisms of mast cells, their immunomodulation capacity, and their proangiogenic potential will open new perspectives regarding the development of future therapeutic strategies targeted towards these multifunctional cells.

## Abbreviations

bFGF: Basic fibroblast growth factor
CTMCs: Connective tissue mast cells
IL: Interleukin
INF-*γ*: Interferon-*γ*
LT: Leukotriene
MCD: Mast cell density
MCs: Mast cell
MVD: Microvessels density
MIF: Macrophage inhibitory factor
MMCs: Mucosal mast cells
MMP: Matrix metalloproteinase
PAF: Platelet activating factor
PAR: Protease activated receptors
RANTES: Regulated upon activation normal T cell expressed and secreted
SCC: Squamous cell carcinomas
SP: Substance P
TGF*β*: Transforming growth factor *β*
TNF: Tumor necrosis factor
VEGF: Vascular endothelial growth factor.
